# The significance of ERK5 catalytic-independent functions in disease pathways

**DOI:** 10.3389/fcell.2023.1235217

**Published:** 2023-08-04

**Authors:** Nhat-Tu Le

**Affiliations:** Center for Cardiovascular Regeneration, Department of Cardiovascular Sciences, Houston Methodist Research Institute, Houston, TX, United States

**Keywords:** MAPK, ERK5 activation, S496 phosphorylation, SASP, SAS

## Abstract

Extracellular signal-regulated kinase 5 (ERK5), also known as BMK1 or MAPK7, represents a recent addition to the classical mitogen-activated protein kinase (MAPK) family. This family includes well-known members such as ERK1/2, c-Jun N-terminal kinase (JNK), and p38 mitogen-activated protein kinase (p38 MAPK), as well as atypical MAPKs such as ERK3, ERK4, ERK7 (ERK8), and Nemo-like kinase (NLK). Comprehensive reviews available elsewhere provide detailed insights into ERK5, which interested readers can refer to for in-depth knowledge (Nithianandarajah-Jones et al., 2012; Monti et al., Cancers (Basel), 2022, 14). The primary aim of this review is to emphasize the essential characteristics of ERK5 and shed light on the intricate nature of its activation, with particular attention to the catalytic-independent functions in disease pathways.

## 1 General information

### 1.1 Structure

**FIGURE 1:**
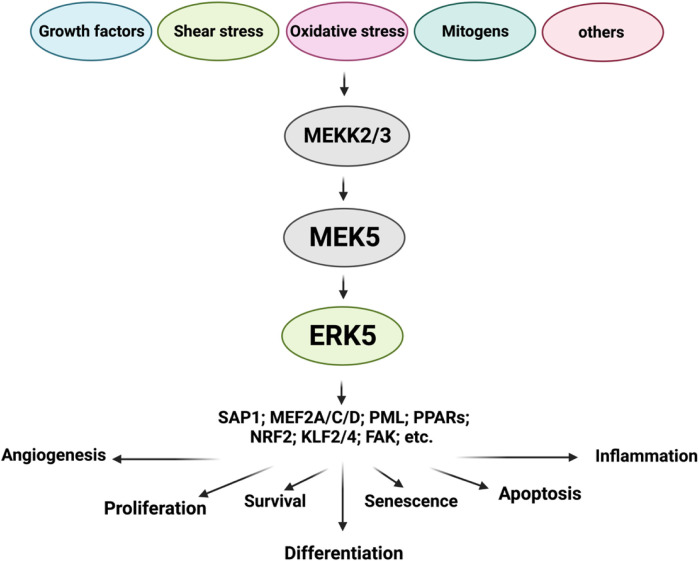
ERK5 upstream regulators and substrates (Figure created using BioRender).

ERK5 is a multifunctional molecule (Zhou et al., 1995; Yang et al., 1998; Wagner et al., 2009; Cargnello and Roux, 2011; Le et al., 2013; Soares-Silva et al., 2016; Zou et al., 2019; Zhao et al., 2022), consisting of two main domains: the NH2-terminal kinase domain (KD) and the large COOH-terminal transcriptional activation domain (TAD) (Wang and Tournier, 2006; Drew et al., 2012; Lochhead et al., 2012; Nithianandarajah-Jones et al., 2012; Le et al., 2013; Le et al., 2014). The NH2-terminal KD shares approximately 50% identity with ERK1/2. Within the COOH-terminal TAD, there is a nuclear localization signal (NLS) domain (amino acids 505-539) and two TADs (amino acids 664-789) (Kasler et al., 2000; Akaike et al., 2004). The NH2-terminal KD of ERK5 contains several distinct regions. These include the cytoplasmic targeting domain (amino acids 1-77), responsible for directing ERK5 to the cytoplasm. The MEK5 binding domain (amino acids 78-139) facilitates interaction with its upstream activator, MEK5. The oligomerization domain (amino acids 140-406) promotes the formation of ERK5 protein complexes. Additionally, the conserved domain (amino acids 350-358) is also present in this region. Furthermore, the NH2-terminal KD features the T-E-Y phosphorylation motif (MEK5 phosphorylation sites) located at amino acids 218-220. Phosphorylation of this motif is crucial for enhancing both the kinase and transcriptional activities of ERK5. Importantly, the ERK5 NH2-terminal KD acts as a positive regulator of the two COOH-terminal TADs, which contribute to ERK5’s transcriptional activation capabilities. In the middle of ERK5, there is the MEF2 interacting domain (amino acids 440-501) (Figure 1) (Akaike et al., 2004).

### 1.2 Phosphorylation residues

In addition to the well-known T-E-Y phosphorylation motif (MEK5 phosphorylation sites) located at amino acids 218-220, specifically phosphorylated at Thr219 and Tyr221, ERK5 can undergo phosphorylation at multiple other residues. These include T28, S421, S433, S496, S731, T733, as well as some unidentified sites (Nithianandarajah-Jones et al., 2012; Monti et al., 2022).

### 1.3 Cellular localization

ERK5 displays a distinct cellular localization compared to other conventional MAPK family members due to its unique autophosphorylation capability within the NLS region. These autophosphorylation events, which are not conserved in other MAPK members, facilitate the nuclear translocation of ERK5. Once inside the nucleus, ERK5 interacts with and activates downstream transcription factors, including SAP1 (Kamakura et al., 1999), c-FOS, c-Myc, Fra-1 (Terasawa et al., 2003), Myocyte enhancer factor-2 A, C, D (MEF2A, C, D) (Yang et al., 1998; Kasler et al., 2000; Potthoff and Olson, 2007; Kim et al., 2008; Gomez et al., 2016), promyelocytic leukemia protein (PML) (Bernardi and Pandolfi, 2007), peroxisome proliferator-activated receptors (PPARs) (Woo et al., 2006), and NF-E2-related factor 2 (NRF2) (Kim et al., 2012). In its quiescent state, the NH2-terminal KD of ERK5 interacts with its COOH-terminal TAD through an intramolecular association. This association keeps ERK5 in a closed conformation in the cytoplasm, thereby inhibiting ERK5 kinase activation (Buschbeck and Ullrich, 2005; Erazo et al., 2020). However, upon MEK5-dependent T-E-Y phosphorylation, the intramolecular association is disrupted. This disruption leads to the phosphorylation of various residues on the COOH-terminal TAD, which exposes the NLS and enables nuclear translocation. Additionally, this process activates ERK5’s transcriptional activity.

### 1.4 Upstream regulators

MEK5 serves as the key upstream activator of ERK5. Various upstream activators have been reported to regulate MEK5 activation. These include RAS, the proto-oncogene tyrosine-protein kinase Src (SRC), TPL/COT, and protein kinase B (AKT) (Barros and Marshall, 2005; Nithianandarajah-Jones et al., 2012). In specific cancers such as neuroblastoma and malignant mesothelioma, the phosphatidylinositol 3-kinase (PI3K/AKT) signaling pathway can activate MEK5, thus initiating the MEK5/ERK5 signaling cascade (Ramos-Nino et al., 2008; Umapathy et al., 2014). In breast cancer, the activation of signal transducer and activator of transcription 3 (STAT3) has been reported to significantly increase the transcription of MEK5 (Liu et al., 2017). In HKC-8 transformed cells and human primary renal proximal tubule epithelial cells (PTECs), the transforming growth factor-beta 1 (TGF-β1) signaling pathway has been shown to mediate MEK5/ERK5 activation by facilitating the binding of ERK5 to MEF2C through the anaplastic lymphoma kinase (ALK) receptor and p38 MAPK (Browne et al., 2008). Furthermore, MEKK2/3, which serves as upstream regulators of MEK5, exhibit activation in various cancer types, including colorectal, prostate, esophageal, breast, cervical, kidney, and lung cancer. Conversely, several phosphatases, such as phosphotyrosine-specific phosphatase (PTP-SL), dual-specificity phosphatases (DUSPs), and protein kinase C (PKC), can inhibit the activation of ERK5, thereby regulating its downstream signaling (Dong et al., 2001; Kendrick and Bogoyevitch, 2007).

### 1.5 Activation

The activation of ERK5 is mediated by its upstream regulator, MEK5 (Soares-Silva et al., 2016). The MEK5/ERK5 signaling pathway can be triggered by a diverse range of stimuli, including mitogens, stressors like cytokines, fluid shear stress, high osmolarity, oxidative stress, and growth factors (Kato et al., 1998) such as vascular endothelial growth factor (VEGF), epidermal growth factor (EGF), nerve growth factor (NGF) (Shao et al., 2002), granulocyte colony-stimulating factor (G-CSF) (Dong et al., 2001), fibroblast growth factor (FGF) (Kesavan et al., 2004), and platelet-derived growth factor (PDGF) (Kato et al., 1997; Hayashi et al., 2004; Kesavan et al., 2004; Rovida et al., 2008). Inflammatory cytokines, including interleukin-6 and -8 (IL-6 and IL-8) (Carvajal-Vergara et al., 2005), as well as osmotic stress (Abe et al., 1996), oxidative stress, and shear stress (Le et al., 2013; Le et al., 2014; Monti et al., 2022) also contribute to the activation of MEK5/ERK5 signaling. Upon activation, the inhibitory effects regulated by the intramolecular association of ERK5 within the NH2-terminal KD are relieved, leading to the activation of its COOH-terminal TAD. The interaction between the ERK5 NH2-terminal KD and the ERK5 COOH-terminal TADs, along with transcription factors such as MEF2 (Kato et al., 1997), PPARγ (Akaike et al., 2004), and NRF2 (Kim et al., 2012), is necessary for ERK5’s transcriptional activity. The activated MEK5/ERK5 signaling pathway participates in various vital biological functions, including proliferation, apoptosis, differentiation, survival, and regulation of cell fate.

### 1.6 Substrates

ERK5, functioning as a kinase with a TAD, regulates numerous molecules. Here, I highlight a few of its substrates:

(i) Sap1 (Kamakura et al., 1999): ERK5 phosphorylation of Sap1 enhances its transcriptional activity through the Serum Response Element (SRE), leading to increased expression of c-FOS (Kamakura et al., 1999).
(ii) Fra-1 (Terasawa et al., 2003): Co-expression of ERK5 with catalytically active MEK5 (CA-MEK5) in Cos-7 cells enhances the phosphorylation, stabilization, and transcriptional activity of c-FOS and Fra-1 (Terasawa et al., 2003).
(iii) MEF2A, C, and D (Yang et al., 1998; Kasler et al., 2000; Potthoff and Olson, 2007; Kim et al., 2008; Gomez et al., 2016): ERK5 interacts with MEF2C through binding of the ERK5 C-terminal domain (amino acids 440-501) to the N-terminal end of MEF2 (Yang et al., 1998; Kasler et al., 2000). ERK5 activation by serum or EGF promotes MEF2A, C, and D transcriptional activity. The dominant negative mutant of ERK5 abolishes the EGF-induced activation of MEF2A and D^42^.
(iv) PML (Bernardi and Pandolfi, 2007): ERK5 interacts with PML at the nuclear bodies of cancer cells, resulting in the phosphorylation of PML at S403 and T409. This phosphorylation disrupts the PML-MDM2 interaction, impairs PML-mediated p21 activation, and downregulates p53 expression, thereby inhibiting the tumor suppressor activity of PML (Yang et al., 2010).
(v) PPARs (Woo et al., 2006): Activation of the MEK5/ERK5 signaling pathway enhances PPARδ transcriptional activity in C2C12 cells, as reported by Woo et al. (2006). This indicates that ERK5 TAD activity is required for the coactivation of PPARδ by ERK5. Furthermore, laminar flow-mediated activation of the MEK5/ERK5 pathway also increases PPARγ transcriptional activity, contributing to the suppression of pro-inflammation (Woo et al., 2008; Clark et al., 2011).
(vi) NRF2 (Kim et al., 2012): ERK5 is involved in the activation of NRF2 induced by laminar flow. ERK5 depletion or inhibition by specific RNA or pharmacological inhibitors, respectively, hampers laminar flow-mediated NRF2-dependent gene expressions. Conversely, ERK5 activation enhances NRF2 transcriptional activity and its nuclear translocation (Kim et al., 2012). By activating NRF2, ERK5 prevents oxidative stress-induced cytotoxicity effects.
(vii) KLF2,4 (Woo et al., 2008; Clark et al., 2011): Laminar flow activates the MEK5/ERK5 signaling pathway, leading to increased KLF2,4 transcriptional activity and endothelial nitric oxide synthase (eNOS) expression, contributing to the maintenance of vascular homeostasis through anti-inflammatory effects (Woo et al., 2008; Clark et al., 2011)**.**

### 1.7 SUMOylation

In addition to being phosphorylated at various residues, ERK5 can undergo SUMOylation at the Lysine 6/22 (K6/22) residue in the NH2-teminal domain. Anti-inflammatory stimuli, such as laminar flow, activate PPARγ and KLF2 by phosphorylating ERK5 at the T-E-Y phosphorylation motif. This activation leads to the inhibition of adhesion molecule expression and exerts anti-inflammatory effects in endothelial cells (ECs). On the other hand, pro-inflammatory stimuli like H_2_O_2_ and AGEs increase ERK5 K6/22 SUMOylation, which represses ERK5/MEF2 transcriptional activity, KLF2 promoter activity, and the expression of KLF2 and eNOS induced by laminar flow. It is worth noting that ERK5 SUMOylation represses ERK5 transcriptional activity through a mechanism that is independent of ERK5 kinase activity (Woo et al., 2008; Clark et al., 2011).

## 2 ERK5 in cancer

### 2.1 ERK5 expression and activation: implication in various types of cancer

The MEK5/ERK5 signaling pathway has gained significant attention as a crucial player in the initiation, progression, and metastasis of a diverse array of cancers. Its involvement has been observed in breast cancer, lung cancer, colorectal cancer, prostate cancer, pancreatic cancer, melanoma, ovarian cancer, and glioblastoma. Extensive research has firmly established its association with unfavorable overall outcomes in these maligancies (Montero et al., 2009; Ramsay et al., 2011; Stecca and Rovida, 2019; Monti et al., 2022).

For example, the role of the MEK5/ERK5 pathway in prostate cancer (PCa) cell proliferation has been extensively studied and well-established (Mehta et al., 2003; McCracken et al., 2008; Stecca and Rovida, 2019). Mehta et al. (2003) conducted a study involving immunohistochemistry on 127 cases of PCa and 20 cases of benign prostatic hypertrophy. Their analysis revealed elevated levels of MEK5 expression in PCa compared to benign prostatic tissue. Strong expression of MEK5 was associated with the presence of bony metastases and indicated a less favorable disease-specific survival outcome. Importantly, among patients with a high Gleason score of 8-10, MEK5 overexpression provided additional prognostic value for survival. To explore the functional implications of MEK5, the researchers performed MEK5 transfection experiments, confirming its ability to induce proliferation, motility, and invasion in PCa cells. Furthermore, the study demonstrated that MEK5 expression led to a significant increase in mRNA expression of MMP-9, while no effect was observed on MMP-2. Luciferase reporter assays provided evidence that MEK5 upregulates the −670/MMP-9 promoter. Additionally, electromobility shift assays indicated the involvement of activator protein-1 (AP-1) in the MMP-9 promoter, rather than the NF-kB binding site. These findings suggest that MEK5 plays a role in PCa and is associated with disease progression (Kato et al., 2000). In another study, Ramsay et al. (2011) utilized siRNA-mediated knockdown or the MEK inhibitor PD18435 to modulate ERK5 expression or function in human PCa cells, specifically PC3 and PC3-ERK5 cells (stably transfected with ERK5). The researchers performed a series of *in vitro* assays to evaluate the impact of ERK5 signaling on crucial cellular processes such as proliferation, motility, invasion, and invadopodia formation. To assess the expression levels of matrix metalloproteinases and tissue inhibitors of metalloproteases, quantitative real-time PCR was employed. Additionally, immunohistochemistry was utilized to analyze ERK5 expression in primary and metastatic PCa samples. The results revealed that downregulation or inhibition of ERK5 significantly impaired the motility and invasive capabilities of PC3 cells. In an orthotopic PCa model, enhanced ERK5 signaling significantly promoted the formation of metastases *in vivo*. Furthermore, the forced expression of ERK5 in PC3 cells resulted in increased invadopodia formation. Notably, nuclear ERK5 immunoreactivity was markedly upregulated in metastatic PCa compared to benign prostatic hyperplasia and primary PCa. These findings underscore the critical role of the MEK5-ERK5 signaling pathway in driving invasiveness in PCa.

Sticht et al. (2008) conducted gene expression profiling using a comprehensive whole-transcriptome chip encompassing 35,035 gene-specific 70-mer oligonucleotides. The analysis focused on a set of 35 primary oral squamous cell carcinoma (OSCC) samples. Their investigation uncovered differential expression of 7,390 genes between OSCC tumor samples and oral mucosa. To elucidate the primary biological processes implicated in this collection of tumors, the researchers employed MAPPFinder, a component of GenMAPP version 2.1, to generate a statistically ranked list of molecular signaling pathways. The results underscored notable dysregulation of various cancer-related pathways within the OSCC collection, with the MAPK pathway exhibiting significant alterations. Furthermore, employing tissue microarrays and immunohistochemistry, the researchers conducted additional analyses and observed elevated protein expression of ERK1 (22.8%) and ERK5 (27.4%) in the examined cases. Intriguingly, high expression of ERK5, but not ERK1, was found to be associated with advanced tumor stage and the presence of lymph node metastases, suggesting the involvement of ERK5 in the progression of OSCC.

de Jong et al. (2016) conducted a study demonstrating that the loss of ERK1/2 in intestinal epithelial cells leads to impairments in nutrient absorption, migration, and secretory cell differentiation, while cell proliferation remains unaffected. The researchers also discovered that genetic deletion of ERK1/2 or pharmacological targeting of MEK1/2 results in supraphysiological activation of the ERK5 pathway. Moreover, when both the ERK1/2 and ERK5 pathways were simultaneously targeted, a more potent suppression of cell proliferation was observed in murine intestinal organoids and human colorectal cancer cell lines. These findings suggest that in intestinal epithelial cells, ERK5 acts as a common bypass route that rescues cell proliferation when ERK1/2 signaling is disrupted.

Tusa et al. (2018) investigated the involvement of the ERK5 signaling pathway in melanoma. They consistently observed the expression of ERK5 in human melanoma tissues and noted its activation in melanoma cells. Through genetic silencing and pharmacological inhibition of the ERK5 pathway, the researchers observed a significant reduction in the growth of melanoma cells and xenografts, irrespective of whether they harbored wild-type or mutated BRAF (V600E). The study also revealed that oncogenic BRAF positively regulates the expression, phosphorylation, and nuclear localization of ERK5. It was observed that BRAF enhances both the kinase and transcriptional activity of ERK5. Importantly, the study highlighted the necessity of reducing nuclear ERK5, which plays a critical role in regulating cell proliferation, through combined pharmacological inhibition of BRAFV600E and MEK5. Furthermore, the researchers demonstrated that combining MEK5 or ERK5 inhibitors with the BRAFV600E inhibitor vemurafenib was more effective than using single treatments alone. This combination therapy exhibited improved outcomes in reducing colony formation and inhibiting the growth of BRAFV600E melanoma cells and xenografts. These findings emphasize the significant role of the ERK5 pathway in melanoma growth, both in laboratory settings and in living organisms.

Pereira et al. (2016) conducted a study to explore the influence of the MEK5/ERK5 pathway on the sensitivity of colon cancer cells to 5-fluorouracil (5-FU), a commonly used chemotherapy drug. The researchers observed a correlation between increased expression of ERK5 and poor overall survival in colon cancer patients. When colon cancer cells were exposed to 5-FU, there was a decrease in the expression and/or activation of endogenous KRAS/MEK5/ERK5 signaling. Conversely, constitutive activation of MEK5 reduced the cytotoxic effects of 5-FU. Using genetic and pharmacological approaches, the researchers demonstrated that inhibition of ERK5 increased caspase-3/7 activity and facilitated apoptosis following 5-FU treatment. This effect was associated with enhanced p53-mediated transcriptional activation of p21 and PUMA, which are key regulators of cell cycle arrest and apoptosis. Additionally, the study showed that inhibiting ERK5 sensitized HCT116 p53^+/+^ cells to 5-FU, while HCT116 p53^−/−^ cells remained resistant, suggesting the involvement of a p53-dependent mechanism in mediating 5-FU sensitization. Moreover, in a murine subcutaneous xenograft model, inhibition of ERK5 using XMD8-92 enhanced the antitumor effects of 5-FU. This combination resulted in increased apoptosis and a significant reduction in tumor growth. Furthermore, employing a series of three-dimensional models derived from cell lines to investigate the impact of inhibiting ERK5 on colon cancer stem-like cells. Their study revealed that pharmacological inhibition of ERK5 had a significant suppressive effect on the molecular and functional characteristics of these cells. Specifically, inhibition of ERK5 using XMD8-92 led to a notable reduction in the formation of primary and secondary spheres, as well as downregulation of pluripotency transcription factors SOX2, NANOG, and OCT4. Additionally, the proportion of tumor cells exhibiting elevated ALDH activity, a marker associated with stem-like properties, was decreased upon ERK5 inhibition. Importantly, the researchers found that inhibiting ERK5 also enhanced the sensitivity of colon cancer cells to chemotherapy utilizing 5-FU. Mechanistically, they observed that ERK5 inhibition resulted in decreased expression of IL-8 and attenuated NF-κB transcriptional activity. This suggests the existence of an ERK5/NF-κB/IL-8 signaling axis that regulates the malignant behavior of stem-like cells in colon cancer (Pereira et al., 2019).

Sanchez-Fdez et al. (2019) conducted a study aimed at assessing the activation and expression of MEK5 and ERK5 proteins in lung adenocarcinoma (LUAD) samples and their corresponding non-tumoral counterparts. The researchers observed the presence of constitutively active MEK5, as indicated by the presence of phosphorylated MEK5, exclusively in the tumor samples. They also detected phosphorylated ERK5 solely in the LUAD samples. Additionally, the expression levels of both MEK5 and ERK5 were found to be higher in the tumor samples compared to the non-tumoral counterparts. To further investigate the clinical implications of MEK5 and ERK5 expression, the researchers analyzed pooled data from the LUAD cohort using the publicly available KM-Plotter database. This analysis revealed a significant association between high levels of combined MEK5 and ERK5 expression and poor overall survival in lung cancer patients. Notably, patients with high MEK5/ERK5 expression exhibited a significantly shorter median survival time of 69 months, compared to those with low MEK5/ERK5 expression, who had a median survival time of 112.67 months. These findings underscore the involvement of the MEK5-ERK5 pathway in human lung cancer (Sanchez-Fdez et al., 2019; Sanchez-Fdez et al., 2021).

Extensive clinical evidence strongly supports the dysregulation of ERK5 in human breast cancer. Hoang et al. (2020) conducted a study using the CRISPR/Cas9 system to deplete ERK5 expression in MDA-MB-231 and Hs-578T cells. The results demonstrated that ERK5 depletion led to the loss of mesenchymal features, evident from changes in gene expression profiles and cell morphology. Moreover, ERK5 depletion suppressed triple-negative breast cancer (TNBC) cell migration. *In vivo* xenograft experiments revealed that ERK5 knockout disrupted tumor growth kinetics, which could be restored by using a high concentration of Matrigel™. Additionally, ERK5 knockout led to a reduction in the expression of the angiogenesis marker CD31, suggesting ERK5’s involvement in angiogenesis. RNA-sequencing analyses demonstrated the downregulation of matrix-associated genes, integrins, and pro-angiogenic factors in ERK5 knockout cells. Further investigations using tissue decellularization combined with cryo-scanning electron microscopy and biomechanical property analysis indicated that ERK5 knockout resulted in the loss of extracellular matrix (ECM) fiber alignment and mechanosensing capabilities in breast cancer xenografts compared to parental wild type (WT) cells. In another study conducted by Xu et al. (2021), *in vitro* experiments highlighted the significant involvement of ERK5 in maintaining the invasive capabilities of TNBC cells through the activation of focal adhesion protein kinase (FAK). Specifically, the researchers demonstrated that the kinase-independent function of ERK5 plays a critical role in phosphorylating FAK at Tyr397. To further investigate the implications of ERK5, the researchers silenced ERK5 in mammary tumor grafts, which resulted in the disruption of FAK phosphorylation at Tyr397. Notably, this intervention effectively suppressed TNBC cell metastasis to the lung without affecting tumor growth. These findings indicate a functional relationship between ERK5 and FAK signaling in promoting malignancy, particularly in the context of breast cancer (Sato et al., 2005; Hoang et al., 2017; Paudel et al., 2021; Xu et al., 2021).

Recently, Arconada-Luque et al. (2022) conducted a study using a murine 3-methyl-cholanthrene (3MC)-induced sarcoma model and observed the development of pleomorphic sarcomas with muscle differentiation, accompanied by an upregulation of ERK5 expression. Interestingly, this upregulation of ERK5 was also observed in human sarcomas of muscular origin, including leiomyosarcoma and rhabdomyosarcoma. To further explore the role of ERK5 in sarcoma, the researchers utilized cell lines derived from the 3MC-induced tumors. They employed specific shRNAs to deplete the expression of ERK5 and found that this depletion resulted in decreased *in vitro* growth, reduced colony-forming capacity, and a substantial inhibition of tumor growth *in vivo*. Notably, transcriptomic profiling of the ERK5-abrogated cell lines using RNA-sequencing revealed a deregulated gene expression pattern associated with key biological processes, including angiogenesis, migration, and motility. Importantly, these alterations in gene expression correlated with improved prognostic outcomes in human sarcoma pathology. Among the differentially expressed genes, the researchers identified KLF2 as a critical mediator of the biological effects of ERK5. By specifically interfering with KLF2, they demonstrated its importance in the ERK5-mediated effects, highlighting the significance of the ERK5-KLF2 axis as a crucial determinant of sarcoma biology. These findings provide compelling evidence for the involvement of the ERK5-KLF2 axis in sarcoma.

Dieguez-Martinez et al. (2022) conducted a study investigating the role of ERK5 in endometrial cancer. Analysis of the PanCancer Atlas dataset revealed alterations in components of the MEK5-ERK5 pathway in 48% of patients with this disease. The researchers found that inhibiting or silencing ERK5 led to a decrease in EGF-induced proliferation of endometrial cancer cells. Furthermore, genetic deletion of MEK5 resulted in impaired proliferation of endometrial cancer cells and reduced tumor growth capacity in nude mice. In both endometrial cancer cells and xenografts, pharmacological inhibition or silencing of ERK5 impaired the NF-kB pathway. Additionally, a positive correlation was observed between ERK5 levels and p65/RELA protein levels in human endometrial cancer tumor samples. Mechanistically, the researchers discovered that genetic or pharmacological impairment of ERK5 led to downregulation of NEMO/IKKγ expression, which subsequently resulted in reduced activity of p65/RELA and induced apoptosis in both endometrial cancer cells and xenografts. Importantly, the impaired apoptosis was rescued by overexpressing NEMO/IKKγ. Notably, inhibition of ERK5, deletion of MEK5, or inhibition of NF-kB sensitized endometrial cancer cells to the toxic effects of standard endometrial cancer chemotherapy (paclitaxel/carboplatin). Moreover, inhibition of ERK5 in combination with paclitaxel led to reduced growth of tumor xenografts in mice. These findings highlight the critical involvement of the ERK5-NEMO-NF-κB pathway in regulating proliferation and survival of endometrial cancer cells.

### 2.2 ERK5 mediates cancer cell cycle progression and proliferation

ERK5 plays a crucial role in cancer by regulating cancer cell proliferation and cell cycle progression, as evidenced by numerous studies (Kato et al., 1998; Mehta et al., 2003; Stecca and Rovida, 2019). Initial research demonstrated that ERK5 activation is essential for EGF-mediated HeLa cell proliferation (Kato et al., 1997). Subsequent studies have provided compelling evidence supporting ERK5’s regulatory function in cancer cell cycle progression (Kato et al., 1998) and proliferation (Kato et al., 1997). Depletion of ERK5 using RNA interference (siRNA) or pharmacological inhibition of ERK5 activity has been shown to inhibit cell cycle progression and proliferation in various cancer types. Notably, ERK5 depletion in cancer cells hinders cancer growth, proliferation, and the expression of proteins involved in angiogenesis (e.g., CD31) and extracellular matrix integrity, thereby influencing cancer biology (Sato et al., 2005; Hoang et al., 2017; Paudel et al., 2021).

ERK5 inhibition prevents cells from entering the S phase of the cell cycle (Kato et al., 1998) by stabilizing cyclin-dependent protein kinase inhibitors, such as p21 and p27 (Perez-Madrigal et al., 2012). In human breast cancer MDA-MB-231 cells, activation of ERK5 triggers c-Myc-dependent transcriptional activation of miR-17-5p and miR-20a, resulting in the inhibition of p21 mRNA translation (Perez-Madrigal et al., 2012). Notably, ERK5 regulates mitogenic signals, including cyclin D1, SGK c-MYC, n-MYC, p90RSK2, and NF-κB (Kato et al., 1997; Hayashi et al., 2001; Mulloy et al., 2003; Hayashi et al., 2005; Garaude et al., 2006; Cude et al., 2007; Umapathy et al., 2014), thereby influencing cell cycle progression. Specifically, ERK5 activation controls the G2/M cell cycle progression (Cude et al., 2007; Yang and Lee, 2011; Song et al., 2017; Pereira and Rodrigues, 2020) and entry into mitosis (Cude et al., 2007). Increased ERK5 activity raises the mitotic index (Cude et al., 2007; Girio et al., 2007) through the activation of the transcription factor NF-kB (Girio et al., 2007), which enhances the expression of mitosis-related genes such as cyclins B1 and B2 and phosphatase 2 (CDC25B). Moreover, ERK5 regulates the G1/S transition by modulating cyclin D1 expression (Mulloy et al., 2003). Additionally, ERK5 prevents caspase activation by binding to and inactivating the pro-apoptotic protein Bim (Girio et al., 2007).

Research efforts have been dedicated to developing ATP-competitive ERK5 inhibitors; however, these inhibitors have been unable to fully replicate the effects observed with genetic loss of ERK5. This discrepancy suggests that ERK5 may have significant catalytic-independent functions. In order to explore the potential catalytic-independent functions of ERK5. You et al. (2022) introduced a potent and selective heterobifunctional degrader of ERK5 known as INY-06-061. Intriguingly, the degradation of ERK5 induced by INY-06-061 did not lead to anti-proliferative effects in multiple cancer cell lines, nor did it suppress inflammatory responses in ECs, in contrast to the outcomes observed with genetic knockdown of ERK5. These findings emphasize the complex functions of ERK5 and highlight the necessity of further exploring its catalytic-independent activity.

### 2.3 ERK5 mediates cancer cell survival, differentiation, and angiogenesis

ERK5 plays a significant role in cell survival, differentiation, and angiogenesis, and comprehensive information on these aspects can be found in detailed reviews elsewhere (Wang and Tournier, 2006; Drew et al., 2012; Lochhead et al., 2012; Nithianandarajah-Jones et al., 2012). In estrogen receptor-positive (ER^+^) breast cancer cells, hyperactivation of MEK5 promotes tumorigenesis through an estrogen-independent mechanism. Notably, ERK5 activation induces epithelial-to-mesenchymal transition, contributing to drug resistance and metastasis. Depletion of ERK5 using CRISPR/Cas9 in MDA-MB-231 and Hs-578T cells results in the loss of mesenchymal features, as observed through changes in gene expression profiles and cell morphology and suppresses the migration of TNBC cells. Moreover, in mouse models, ERK5 depletion in tumor-associated macrophages inhibits the growth of melanoma and lung carcinoma (Giurisato et al., 2020), while its depletion in keratinocytes prevents inflammation-promoted tumorigenesis (Wu et al., 2015). ERK5 also plays a role in maintaining the activation of survival signaling pathways (Liu et al., 2003) and regulates the G1/S transition by suppressing the CDK inhibitor p21 and increasing the expression of cyclin D1 (Yang et al., 2010; Yang and Lee, 2011; Song et al., 2017; Pereira and Rodrigues, 2020), thereby promoting cell cycle progression. Given its involvement in these critical processes, ERK5 has emerged as a promising therapeutic target for inhibiting cancer progression and proliferation (Mehta et al., 2003; Ramsay et al., 2011; Benito-Jardon et al., 2019; Monti et al., 2022). Researchers have recognized the potential of targeting ERK5 signaling to develop novel anti-cancer strategies aimed at improving treatment outcomes.

### 2.4 ERK5 mediates cancer-associated inflammation

Accumulating evidence have also highlighted the involvement of ERK5 activation in cancer-associated inflammation. Inhibition of ERK5 in macrophages induces a transcriptional switch that impedes the polarization of protumor macrophages (Giurisato et al., 2018). In skin carcinogenesis, ERK5 regulates the expression of various proinflammatory cytokines, and inhibition of ERK5 prevents inflammation-associated tumorigenesis (Finegan et al., 2015). In a 12-O-tetradecanoylphorbol-13-acetate (TPA)-promoted two-stage skin carcinogenesis model, ERK5 is essential for the induction of IL-1α, IL-1β, and COX-2, while its inhibition has no effect on TNFα and IL-6 (Finegan et al., 2015). Additionally, an unbiased mass spectrometry-based secretome analysis conducted in lung cancer cells has revealed that ERK5 is crucial for IL-6 production in cancer cells, and inhibiting or depleting ERK5 prevented this observed phenotype (Riegel et al., 2021).

Metastasis, the leading cause of cancer-related mortality, involves a complex process in which tumor cells and the tumor microenvironment collaborate to release circulating tumor cells (CTCs) into the bloodstream. These CTCs survive in circulation, extravasate, and establish secondary metastatic sites, significantly impacting metastatic outcomes. Reprogramming of the transcriptomic landscape is a characteristic feature of metastasis. However, identifying the key regulators that drive pathological gene expression remains challenging, especially in childhood cancer. In a study conducted by Green et al. (2020), whole tumor and single-cell RNA sequencing were employed in primary bone cancer and CTCs. The researchers utilized weighted gene co-expression network analysis to identify coordinated changes in metastatic gene expression. By applying this approach to data obtained from cell line models, clinical samples, and xenograft mouse models, the study revealed the crucial role of ERK5/MMP-9 signaling as a driver of metastasis in primary bone cancer. Knockdown of ERK5 using siRNA resulted in a reduction in proliferation, colony formation, migration, tumor growth, macrophage residency/polarization, and lung metastasis. Additionally, a decrease in the expression of activated interleukins IL-1α, IL-6, IL-8, as well as mesenchymal markers VIM and VEGF, was observed upon ERK5 loss. These findings strongly suggest the involvement of the ERK5/MMP-9 axis as a central signaling hub in the metastasis of primary bone cancer.

Tumors employ diverse strategies to suppress antitumor immune responses. In their study, Riegel et al. (2021) conducted an unbiased analysis using mass spectrometry-based secretome analysis in lung cancer cells to identify factors secreted by tumor cells. Among the factors identified, IL-6 emerged as a prominent factor secreted by tumor cells as well as cancer-associated fibroblasts derived from cancer patients. The researchers investigated the impact of tumor cell supernatants on dendritic cell (DC) cultures and observed a significant inhibition of IL-12p70 production, while other activation markers on the cell surface remained unaffected. However, this inhibition was reversed when DC cultures were treated with an IL-6 antibody. The impaired production of IL-12p70 in DCs resulted in a compromised differentiation of Th1 cells, but not Th2 or Th17 cells, from naïve CD4^+^ T cells. Furthermore, these findings highlight the crucial role of ERK5 in IL-6 production by tumor cells. They observed that inhibiting ERK5 activity or depleting ERK5 expression prevented IL-6 production in tumor cells.

## 3 ERK5 in cardiovascular disease (CVD)

### 3.1 ERK5’s anti-inflammatory effects in ECs and myeloid cells (MCs)

ERK5 is well-known for its mechanoreceptive functions in ECs, where it plays a pivotal role in transmitting vasoprotective effects induced by laminar blood flow. Beyond its significance in ECs, ERK5 serves as a vital regulator in mechanically stressed tissues such as bone, cartilage, and muscle, contributing significantly to cell survival and differentiation. While ERK5 is involved in various physiological roles, it has also been implicated in numerous diseases, with cancer being a prominent focus of recent studies, as discussed in Section 2. Notably, in the context of cancer, ERK5 has emerged as a critical survival mechanism for tumor cells, helping them withstand the stress induced by anticancer drugs, thereby promoting cancer progression (Kato et al., 1998; Mehta et al., 2003; Montero et al., 2009; Ramsay et al., 2011; Stecca and Rovida, 2019; Monti et al., 2022; Abe et al., 2023). Moreover, ERK5’s dysfunction has been associated with the development of CVD, particularly atherosclerosis (Heo et al., 2014; Paudel et al., 2021).

Under the influence of laminar blood flow conditions, the kinase activity of ERK5 in ECs becomes activated, playing a pivotal role in averting EC inflammation, dysfunction, and apoptosis. Consequently, ERK5 protects the vasculature from pathological conditions and maintains vascular homeostasis, providing crucial support for overall cardiovascular health (Berk, 2008; Le et al., 2013). In our high-throughput screening, we made a significant discovery that certain statins (pitavastatin, simvastatin, and rosuvastatin) and antimalarial drugs (chloroquine, hydroxychloroquine, and quinacrine) can activate both the kinase and transcriptional activity of ERK5. Particularly, pitavastatin induced ERK5’s transcriptional activity and the expression of KLF2 in ECs, and these effects were abolished when ERK5 was depleted. Furthermore, our studies demonstrated that chloroquine and hydroxychloroquine enhance ERK5 kinase activity and inhibit VCAM-1 expression via a mechanism that depends on ERK5 but operates independently of the MEK5 and KLF2/4 signaling pathway (Le et al., 2014). The NRF2 transcription factor participates in regulating antioxidant and cytoprotective genes by interacting with and activating promoters containing the antioxidant response element (ARE) (Mahmoud et al., 2017). Notably, research by Kim et al. revealed that ERK5 kinase activation promotes the binding of ERK5 to NRF2, thereby enhancing NRF2-ARE transcriptional activity (Gorbachev et al., 2007; Kubben et al., 2016). Importantly, we observed that in MCs, NRF2 transcriptional activation inhibits the induction of the senescence-associated secretory phenotype (SASP), suggesting the anti-inflammatory effects of ERK5 kinase activation (Gorbachev et al., 2007; Berk, 2008; Le et al., 2013; Kubben et al., 2016; Singh et al., 2019).

Efferocytosis, the process by which phagocytic cells remove dead and dying cells, is believed to play a crucial role in limiting the progression of atherosclerosis. In a study by Heo et al. (2014), the researchers investigated the underlying mechanism of this process. They discovered that when macrophages were exposed to apoptotic cells or treated with pitavastatin (a statin) *in vitro*, the efferocytosis-related signaling and phagocytic capacity were significantly enhanced in an ERK5 kinase activity-dependent manner. To further validate the significance of ERK5 in efferocytosis, the researchers utilized macrophages isolated from mice with specific ERK5 knockout (KO) in macrophages. These ERK5-deficient macrophages demonstrated reduced efferocytosis capability and showed lower levels of gene and protein expression of molecules involved in efferocytosis. To assess the impact on atherosclerosis, the researchers crossed these ERK5-KO mice with mice lacking the low-density lipoprotein receptor (LDLR) and fed them a high-cholesterol diet. The results revealed that the atherosclerotic plaque formation was accelerated in these mice, and the plaques displayed a more advanced and vulnerable morphology. These findings underscore the central role of ERK5 in enhancing macrophage efferocytosis, which in turn suppresses the formation of atherosclerotic plaques.

### 3.2 Inflammatory effects of ERK5 S496 phosphorylation in ECs and MCs

**FIGURE 2:**
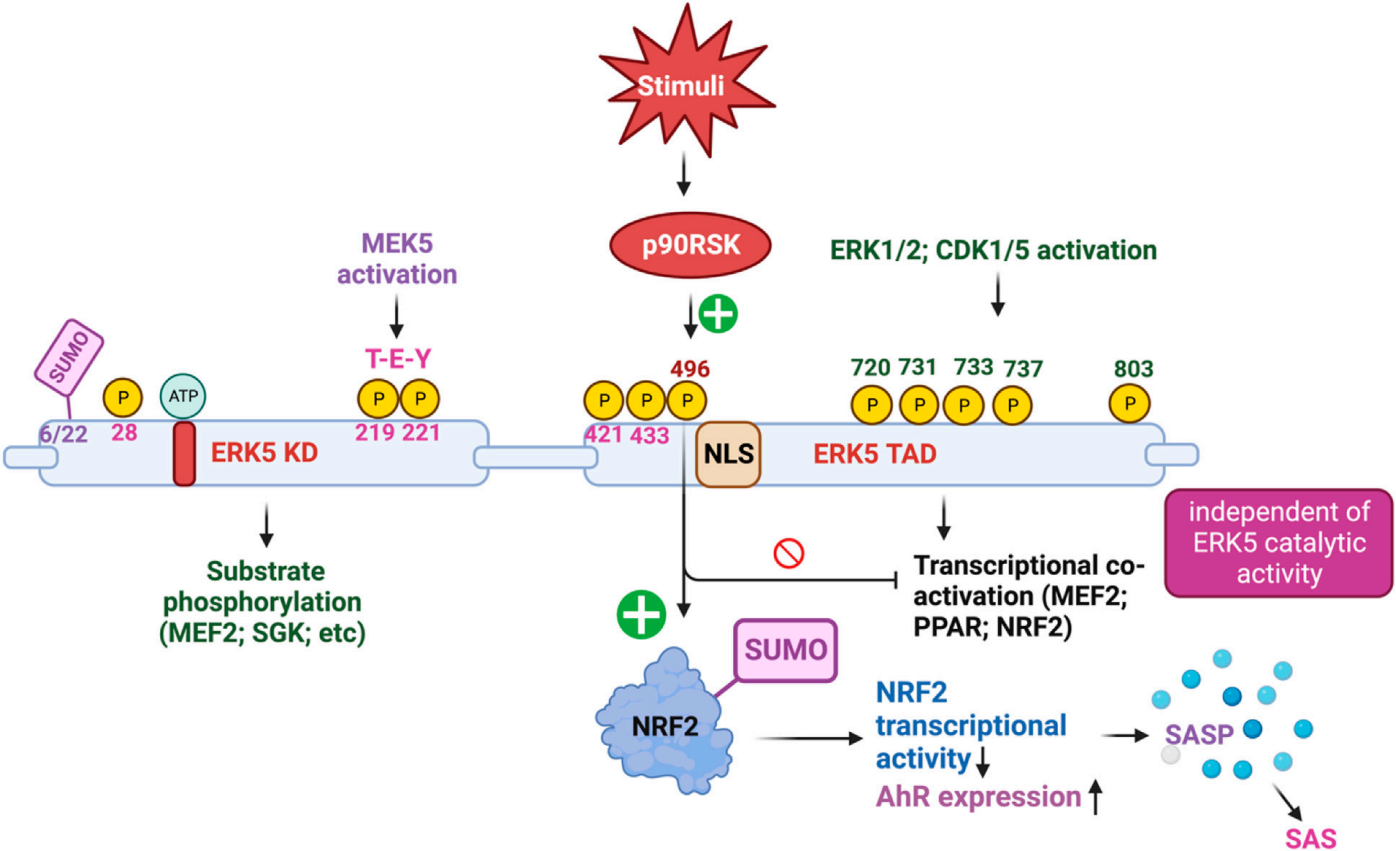
The complex mechanism of action of ERK5 activation (Figure created using BioRender).

To explore the functional role of ERK5 in ECs during vascular inflammation and the formation of atherosclerotic plaques, we utilized inducible EC-specific ERK5-LDLR KO mice. The findings revealed that these mice exhibited elevated leukocyte rolling, impaired vessel reactivity, and increased atherosclerotic plaque formation. An important interaction was observed between ERK5 and the serine/threonine kinase p90RSK, known for its critical role in heart failure (Takeishi et al., 1999; Takeishi et al., 2002; Itoh et al., 2005a; Le et al., 2012). Upon activation, p90RSK interacted with ERK5 and inhibited its transcriptional activity, leading to an upregulation of VCAM-1 expression. This interaction was mediated by p90RSK binding to the COOH-terminal TAD of ERK5 (amino acids 571-807). Additionally, p90RSK directly phosphorylated ERK5 at S496, resulting in the inhibition of ERK5’s transcriptional activity and reduced eNOS expression (Le et al., 2013). Notably, the activation of ERK5’s catalytic function occurs through an ERK5 kinase activity-dependent mechanism (Pearson et al., 2001), whereas ERK5 S496 phosphorylation is driven by an ERK5 kinase activity-independent mechanism (Le et al., 2013; Singh et al., 2019). Furthermore, in diabetic mouse vessels, p90RSK activity was found to be elevated. By employing the specific p90RSK inhibitor, fluoromethyl ketone-methoxyethylamine (FMK-MEA), we were able to ameliorate EC-leukocyte recruitment and improve vascular reactivity in diabetic mice. Interestingly, these beneficial effects of FMK-MEA on leukocyte rolling and vessel reactivity were resistant in EC-specific ERK5 KO mice, highlighting the critical role of EC ERK5 in mediating the inhibitor’s salutary effects on EC dysfunction. Moreover, we discovered that p90RSK-induced ERK5 S496 phosphorylation inhibits NRF2-ARE transcriptional activity through a mechanism independent of both ERK5 kinase activation and ERK5 transcriptional activation. Our findings indicated that p90RSK-induced ERK5 S496 phosphorylation contributed to the development of atherosclerosis (Paez-Mayorga et al., 2018; Singh et al., 2019). Our findings revealed that p90RSK-induced ERK5 S496 phosphorylation does not inhibit ERK5 T-E-Y phosphorylation or ERK5 transcriptional activity, even in the absence of CA-MEK5 expression, which corresponds to a constitutively active form of MEK5. In addition to these significant findings, we made a novel discovery that ERK5 S496 phosphorylation induces NRF2 SUMOylation at a previously unidentified K518 site. This SUMOylation process leads to the inhibition of NRF2 transcriptional activity (see Figure 2). Importantly, this process does not affect ERK5’s catalytic activity but instead contributes to the senescence-associated secretory phenotype (SASP) induced by oxidized low-density lipoprotein (oxLDL) (Singh et al., 2019).

### 3.3 CD36/ERK5 signaling drives oxLDL-mediated platelet caspase activation

Dyslipidemia, a risk factor for thrombotic events, is characterized by increased platelet reactivity facilitated by the scavenger receptor CD36, which recognizes circulating oxidized lipids. CD36 activates redox-sensitive signaling molecules, including ERK5, to promote thrombosis. However, the downstream events of platelet ERK5 activation remain unclear. In their study, Yang et al. demonstrated that oxLDL exposure promotes the exposure of procoagulant phosphatidylserine (PSer) on the surface of platelets. Pharmacological inhibition studies reveal that oxLDL-induced PSer exposure, mediated by CD36 interaction, requires apoptotic caspases as well as downstream signaling molecules including Src kinases, hydrogen peroxide, and ERK5. Caspases play a crucial role in promoting PSer exposure, leading to the recruitment of the prothrombinase complex and subsequent fibrin generation through thrombin activation. Notably, platelet stimulation with oxLDL induces caspase activity, which can be prevented by inhibiting CD36 and ERK5. Furthermore, oxLDL enhances convulxin/glycoprotein VI-mediated fibrin formation by platelets, and this effect can be attenuated by inhibiting CD36, ERK5, and caspases. *In vivo* arterial thrombosis models using apoE-KO hyperlipidemic mice demonstrate increased arterial fibrin accumulation upon vessel injury. Importantly, platelets lacking ERK5 or mice lacking CD36 show decreased fibrin accumulation in high-fat diet-fed conditions comparable to that observed in animals fed a chow diet. These findings suggest that platelet signaling through CD36 and ERK5 induces a procoagulant phenotype in the hyperlipidemic environment by enhancing caspase-mediated-PSer exposure.

## 4 Studies using ERK5 inhibitors have revealed ERK5 complex mechanism of action

The increased activation and expression of ERK5 have been implicated in the pathophysiology of a wide range of cancers (Sanchez-Fdez et al., 2021), suggesting the potential therapeutic value of depleting or pharmacologically inhibiting ERK5 in inflammation and tumorigenesis. To target this pathway, numerous ATP-competitive inhibitors targeting ERK5 catalytic activity have been developed (Wang and Tournier, 2006; Drew et al., 2012; Lochhead et al., 2012; Nithianandarajah-Jones et al., 2012; Le et al., 2013; Le et al., 2014; Cook et al., 2020) and evaluated for their potential in treating cancer and inflammatory diseases (Drew et al., 2012; Cook et al., 2020). However, these inhibitors have not fully replicated the effects observed with genetic loss of ERK5 (Lin et al., 2016), suggesting that ERK5 may have significant catalytic-independent functions. In an effort to explore the potential catalytic-independent functions of ERK5, You et al. (2022) introduced a potent and selective heterobifunctional degrader of ERK5 known as INY-06-061. Intriguingly, the degradation of ERK5 induced by INY-06-061 did not lead to anti-proliferative effects in multiple cancer cell lines, nor did it suppress inflammatory responses in primary ECs. These outcomes contrasted with the effects observed with genetic knockdown of ERK5, indicating that ERK5’s functions are more complex than solely its kinase activity (Lin et al., 2016; You et al., 2022). These findings shed light on the multifaceted nature of ERK5 signaling and suggest that understanding ERK5’s catalytic-independent functions may uncover additional opportunities for developing effective anti-cancer and anti-inflammatory strategies.

In a study conducted by Lin et al. (2016), using the first generation of ERK5 inhibitors (XMD8-92), it was discovered that XMD8-92 has off-target effects on bromodomain-containing proteins (BRDs) and exhibits anti-inflammatory and anti-proliferative activities. However, Lin et al. also developed a highly selective ERK5 inhibitor called AX15836, which displayed no anti-inflammatory or anti-proliferative effects, despite ERK5 depletion showing such effects. These findings led Lin et al. to propose that ERK5 kinas activation is not essential for cellular immune response and proliferation, and that ERK5 regulates inflammation and proliferation through a mechanism independent of kinase activation.

Our study, published in Circulation (2012), focused on the role of ERK5 in ECs and highlighted the significance of ERK5 T-E-Y phosphorylation, which represents ERK5 kinase activation, in mediating the anti-inflammatory and anti-atherosclerotic effects of laminar blood flow (Le et al., 2013). Additionally, our research demonstrated that ERK5 S496 phosphorylation plays a crucial role in inducing pro-inflammatory and atheroprone effects under disturbed blood flow conditions (Le et al., 2013). These findings underscore the distinct biological consequences resulting from inhibiting ERK5 kinase activation, inhibiting ERK5 S496 phosphorylation, and genetically ablating ERK5 (Takeishi et al., 1999; Takeishi et al., 2002; Akaike et al., 2004; Itoh et al., 2005a; Le et al., 2012; Le et al., 2013; Heo et al., 2014). The study conducted by Lin et al., in 2016 (Lin et al., 2016) aligns with and further supports our observations (Le et al., 2013). Their research using ERK5 inhibitors revealed that the functions of ERK5 in inflammation and proliferation are not solely dependent on its kinase activation. This adds valuable insight into the complex regulatory mechanisms of ERK5 and enhances our understanding of its diverse roles in various cellular processes. The collective findings from both studies contribute to the growing knowledge of ERK5 signaling and its potential implications in cardiovascular and inflammatory diseases.

The study conducted by Lochhead et al. (2020) provided valuable insights into the mechanism of action of selective ERK5 inhibitors, namely, BAY-885 and AX15836. Their research revealed that these inhibitors directly bind to the ERK5 KD, inducing conformational changes that trigger ERK5 nuclear translocation. Consequently, the ERK5 COOH-terminal NLS domain of ERK5 is exposed, paradoxically activating ERK5 TADs and enhancing KLF2 transcriptional activity. It is worth nothing that the impact of ERK5 inhibitors on inflammation or proliferation was not investigated in this study (Lochhead et al., 2020). Nonetheless, the findings of Lochhead et al. align with and support our own research findings (Takeishi et al., 1999; Takeishi et al., 2002; Akaike et al., 2004; Itoh et al., 2005a; Le et al., 2012; Le et al., 2013; Heo et al., 2014), thus further contributing to our understanding of ERK5 signaling.

ERK5 S496 phosphorylation contributes to the development of atherosclerosis (Singh et al., 2019). To investigate this further, we employed CRISPR/Cas9 technology to generate ERK5 S496A knock-in (KI) mice and subsequently characterized the atherosclerosis in these mice (Abe et al., 2023). For plaque phenotyping, we utilized imaging mass cytometry to analyze both homozygous ERK5 S496A KI mice and WT mice. Additionally, we isolated bone marrow-derived macrophages from hypercholesterolemic mice and performed RNA-sequencing, along with various *in vitro* assays like senescence, mitochondrial reactive oxygen species (ROS), inflammation assays, and metabolic extracellular flux analysis. Our findings revealed that atherosclerosis was inhibited in ERK5 S496A KI mice, and that the phosphorylation of ERK5 at S496 is a critical mediator of the SASP and senescence-associated stemness (SAS). These effects were attributed to the upregulation of the aryl hydrocarbon receptor (AHR) in plaques and bone marrow-derived macrophages isolated from hypercholesterolemic mice. Moreover, we discovered that ERK5 S496 phosphorylation induces SUMOylation of NRF2 at a novel K518 site, leading to the inhibition of NRF2’s transcriptional activity. It is noteworthy that this effect does not impact the catalytic activity of ERK5 but rather contributes to the SASP induced by oxLDL. In terms of therapeutic implications, we observed that specific ERK5 kinase inhibitors, AX15836 and XMD8-92, successfully inhibit ERK5 S496 phosphorylation. Interestingly, this inhibition appeared to play a role in the anti-inflammatory effects of these inhibitors, suggesting their potential in treating atherosclerosis. Our study has unveiled a novel mechanism involving the macrophage ERK5-NRF2 axis, which plays a significant role in the development of the SAS through the upregulation of AHR. This mechanism provides a crucial explanation for the paradoxical presence of senescence in proliferative plaques, as it allows MCs to circumvent senescence-induced cell cycle arrest during atherosclerosis formation. Moreover, the identification of the SAS phenotype offers valuable insights into the complex dynamics of cellular senescence within atherosclerotic lesions. These findings deepen our comprehension of atherogenesis at the molecular level and open promising avenues for exploring therapeutic strategies targeting the SAS phenotype (Abe et al., 2023).

## 5 Concluding remarks

We (Le et al., 2013) and others (Takeishi et al., 1999; Takeishi et al., 2002; Akaike et al., 2004; Itoh et al., 2005a; Le et al., 2012; Le et al., 2013; Heo et al., 2014; Lin et al., 2016; Lochhead et al., 2020; You et al., 2022) have shown that both the ERK5 KD and the ERK5 TAD play significant but distinct roles, depending on various factors such as stimuli, cellular context, and cell types. Therefore, when considering the use of anti-ERK5 therapeutic approaches to inhibit inflammation, tumor growth, and proliferation, it is crucial to carefully consider these variables. It is important to note that selective ERK5 inhibitors, genetic ablation of ERK5, and depletion of ERK5 may not produce identical effects. The effects observed in the genetic depletion of ERK5, as reported by Lin et al., could be attributed to the removal of ERK5’s non-catalytic function (Lin et al., 2016), which aligns with the findings from our own studies (Takeishi et al., 1999; Takeishi et al., 2002; Akaike et al., 2004; Itoh et al., 2005a; Le et al., 2012; Le et al., 2013; Heo et al., 2014). Thus, understanding the complex functions and mechanisms of action of ERK5 is essential for the development of effective therapeutic strategies targeting ERK5 signaling.

Inhibition of MEK5 signaling or ERK5 kinase activity has been shown to reduce plasma concentrations of proinflammatory cytokines in mice exposed to Toll-like receptor (TLR) ligands, heat-killed *Staphylococcus aureus*, or undergoing sterile lung ischemia-reperfusion injury. Additionally, inhibition of ERK5 has been found to protect endotoxemic mice from death. However, in contrast to these findings, Wilhelmsen et al. observed pro-inflammatory responses of ERK5 to various microbial TLR2 agonists, IL-1β, and TNF-α in human vascular ECs and monocytes, despite the inhibition of both MEK5/ERK5 signaling and ERK5 kinase activation (Wilhelmsen et al., 2015). On the other hand, our research has shown that ERK5 kinase activation is necessary for shear stress-induced anti-inflammatory effects in ECs, and the formation of atherosclerotic plaques is increased in EC- and MC-specific ERK5 KO mice (Akaike et al., 2004; Le et al., 2013; Heo et al., 2014). These findings collectively suggest that the pro-inflammatory responses of ERK5 observed by Wilhelmsen et al. are independent of its catalytic activity, and the protective effects of MEK5 or ERK5 inhibitors may be attributed to the inhibition of an unidentified phosphorylation residue on the ERK5 molecule.

Our recent research has also uncovered a significant connection between cancer and CVD, which can be attributed to ERK5 S496 phosphorylation (Le et al., 2013; Abe et al., 2023; Banerjee et al., 2023). We have observed the formation of a nucleus-mitochondria positive feedback loop that links mitochondrial dysfunction with nuclear telomere dysfunction, resulting in sustained dysfunctions and their associated biological consequences. This intricate feedback loop is primarily regulated by the signaling pathway involving p90RSK-mediated ERK5 S496 phosphorylation (Le et al., 2013; Kotla et al., 2021), as extensively discussed elsewhere (Le et al., 2012; Le et al., 2013; Vu et al., 2018; Singh et al., 2019; Banerjee et al., 2021; Kotla et al., 2021; Banerjee et al., 2022; Abe et al., 2023). Our key finding stems from evidence that demonstrates the occurrence of late cardiovascular complications in cancer survivors following cancer treatments (Yeh et al., 2004). Notably, treatments such as chemotherapy and radiation therapy have been shown to increase the production of mitochondrial ROS (Perillo et al., 2020), thereby activating the redox-sensitive kinase p90RSK (Abe et al., 2000; Itoh et al., 2005b). Subsequently, the activated p90RSK interacts with ERK5 and phosphorylates ERK5 S496 (Le et al., 2013), initiating the inflammatory signaling cascade of p90RSK-ERK5 S496. This signaling pathway exerts inhibitory effects on the transcriptional activity of ERK5 and NRF2 (Kotla et al., 2021), a master regulator of antioxidant. Consequently, impaired NRF2 transcriptional activity leads to a decrease in the expression of crucial antioxidant molecules, including HO1 and Trx1. These molecules play significant roles in initiating a persistent SASP, characterized by the secretion of pro-inflammatory factors. The sustained induction of SASP is implicated in the development of various pathologies associated with cancer and CVD. In summary, ERK5 S496 phosphorylation acts as a critical mechanism linking cancer and CVD. The nucleus-mitochondria positive feedback loop, regulated by the p90RSK-ERK5 S496 signaling pathway, maintains the dysfunctions observed in both diseases and contributes to the persistent induction of SASP.
